# A Spatiotemporal-Oriented Deep Ensemble Learning Model to Defend Link Flooding Attacks in IoT Network

**DOI:** 10.3390/s21041027

**Published:** 2021-02-03

**Authors:** Yen-Hung Chen, Yuan-Cheng Lai, Pi-Tzong Jan, Ting-Yi Tsai

**Affiliations:** 1 Department of Information Management, National Taipei University of Nursing and Health Sciences, Taipei 112, Taiwan; 2 Department of Information Management, National Taiwan University of Science and Technology, Taipei 106, Taiwan; laiyc@cs.ntust.edu.tw (Y.-C.L.); 403402279@gapp.fju.edu.tw (T.-Y.T.); 3 Department of Applied Informatics, Fo Guang University, Yilan 262, Taiwan; ptjan@gm.fgu.edu.tw

**Keywords:** long short-term memory, ensemble learning, convolutional neural network, link flooding attack

## Abstract

(1) Background: Link flooding attacks (LFA) are a spatiotemporal attack pattern of distributed denial-of-service (DDoS) that arranges bots to send low-speed traffic to backbone links and paralyze servers in the target area. (2) Problem: The traditional methods to defend against LFA are heuristic and cannot reflect the changing characteristics of LFA over time; the AI-based methods only detect the presence of LFA without considering the spatiotemporal series attack pattern and defense suggestion. (3) Methods: This study designs a deep ensemble learning model (Stacking-based integrated Convolutional neural network–Long short term memory model, SCL) to defend against LFA: (a) combining continuous network status as an input to represent “continuous/combination attacking action” and to help CNN operation to extract features of spatiotemporal attack pattern; (b) applying LSTM to periodically review the current evolved LFA patterns and drop the obsolete ones to ensure decision accuracy and confidence; (c) stacking System Detector and LFA Mitigator module instead of only one module to couple with LFA detection and mediation at the same time. (4) Results: The simulation results show that the accuracy rate of SCL successfully blocking LFA is 92.95%, which is 60.81% higher than the traditional method. (5) Outcomes: This study demonstrates the potential and suggested development trait of deep ensemble learning on network security.

## 1. Introduction

Internet of Things (IoT) is becoming of crucial importance in social, corporate, and government activities. The notorious distributed denial-of-service (DDoS) attack, however, launches large-scale unexpected traffic to the target servers to exhaust their resources and keep users from accessing the business IoT services. Sixty percent of service providers in the world have experienced DDoS attacks and suffered from huge economic losses as a consequence. With the evolution of DDoS attack technology, a new type of DDoS attacks, called link flooding attacks (LFA), has emerged. Unlike traditional DDoS, LFA arranges a lot of bots to send valid low-rate traffic to decoy servers and manipulate these servers to generate enormous network traffic flooding into backbone links, which connect between a target IoT network segment and the Internet. All servers in the target area will then be degraded or even cut off from network connectivity [1]. Furthermore, for the purpose of expanding the effectiveness of attacks and ensuring the indistinguishability of attack traffic from legitimate traffic, LFA dynamically attacks different target links during different periods, which is called a spatiotemporal series attack pattern [2]. For example, LFA attack some set of target links for a while and attack the other set of target links at another time–space. In summary, LFA are difficult to defend against due to the two research problems: (1) indistinguishability of the changing LFA characteristics and (2) the spatiotemporal attack pattern, leading to the requirement of developing varying LFA defending methodologies.

Defending against LFA is composed of two steps: detecting and mitigating. First, the detecting step sets rules to observe flow or packet information and to judge whether LFA happen. The conventional defending LFA methodology can be classified into types of flow metric threshold [1,3,4,5,6,7,8] and traceroute packet monitoring [9,10]. The flow metric threshold methods determine whether LFA happen according to specific flow metrics exceeding their corresponding thresholds. On the other hand, in [9,10] the authors attempt to monitor the traceroute packets, which are launched by the attacker to acquire the topology and attacks on target links. The second LFA defending step is to mitigate LFA by rerouting traffic [1,10] or blocking malicious traffic [4,5,8,11,12,13] in order to deter the attack, to relieve the harm, and to recover the attacked network to a normal status. The traffic rerouting methods reroute flows that originally travel through the target links to relieve their congestions. The blocklist (or blacklist) works to collect suspicious flows into a blacklist and drop packets based on this blacklist when the LFA have been detected. These LFA defending methods, however, are heuristic in that they rely on the experience of algorithm designers and cannot reflect the changing attack characteristics of LFA in a timely manner.

To overcome the changing attack characteristics of LFA, numerous artificial intelligence (AI) methodologies [11,12,14,15,16,17,18,19,20,21,22,23,24,25] are introduced to defend against LFA through end-to-end functionality (Input: network status; Output: defending action) without any manual intervention [26,27,28,29,30]. The AI-based methodologies, therefore, reduce the inefficient labor cost, subjective judgment, and self-learning regarding the changing attack characteristics of LFA in a timely manner. The basic idea of the current AI-based methodology is to screenshot the current network status as the input sample, then to apply convolution and pooling operations to extract features of the LFA, and finally to decide the possibility of the current network suffering LFA. However, these AI-based methodologies adopt a simple deep learning architecture that can only focus on LFA detection rather than simultaneously coupling with actions of detecting and mediating the LFA. On the other hand, these methods only use one network status at a certain time slice and neglect the fact that LFA are a continuous attacking action or combination of varying attacking actions in different time–space, since LFA are gradually generated, enhanced, and dynamically switched. Ignoring the spatiotemporal attack pattern as we mentioned at the beginning of this study, the conventional AI-based methods inescapably suffer inaccurate LFA detection and mitigation results. 

This study designs a deep ensemble learning model, called stacking-based integrated CNN-LSTM model (SCL), to defend against LFA. SCL adopts a two-step strategy to defend against LFA; (1) once the System Detector located in SDN (software defined network) controller detects an unusual flow, it then sends an alarm and amplifies the network flow features to each link’s switch, and then (2) each link’s switch uses its own mediator module to decide the defense strategy by using continuous network status as input sample and the amplified network flow features. SCL develops three novel technologies to accomplish the above two-step strategy: (a) SCL combines several continuous network status screenshots as an input sample to represent “continuous or combination attacking action” and to help SCL’s CNN operation to extract the features of spatiotemporal attack LFA pattern, (b) SCL applies long short-term memory (LSTM) to store the collected samples in a short memory queue, and periodically review the current evolved LFA patterns and drop the obsolete ones to ensure the decision accuracy and confidence of the long-term memory, (c) stacking System Detector and LFA Mitigator module instead of only one AI module to overcome the issue that previous AI-based methods cannot couple with LFA detection and mediation at the same time. The stacking methodology is just like the military operations; for example, the System Detector acts as a scout to explore an area to gain information about network flow features. Once the System Detector detects an unusual flow, it then raises the alarm and sends the amplified features to frontline links. Then each link uses its own mediator to evaluate its link is under LFA by using current network status and the detection result as an amplifier.

The technical reasons for SCL picking both CNN and LSTM to defend against LFA in IoT networks are twofold. First, the conventional deep learning approaches, which apply a single constituent learning algorithm, often encounter overfitting problems [31,32,33]. Overfitting is when the model is trained exactly to a particular set of data and unwittingly extracts variation (i.e., the noise), as if that variation represents underlying population structure; in such cases, the model may therefore fail to fit additional data or predict future observations. Second, the conventional deep learning approaches [26,27,28,29,30,34,35,36,37] do not notice that the link flooding attacks in IoT networks (e.g., LFA) is spatiotemporal. LFA flooding contains temporal dependencies (time series attacking) and spatial characteristics (varying attack targets due to changing IoT topology). Ignoring the spatiotemporal LFA features would seriously affect the reliability and performance of the prediction. For example, in [26,27,28,29,30] the authors focus on the spatiality of the data, which means the LFA flowing distribution features, but they ignore the temporal dependencies of the LFA flowing directions. On the other hand, the authors of other studies [34,35,36,37] apply the LSTM model to address considering temporal dependencies, but they pay little attention to the spatiality of the data. SCL, therefore, combines CNN and LSTM, extracts spatiotemporal features of data simultaneously, and attempts to obtain better predictive performance than the constituent learning algorithms alone.

This study is organized as follows: Section 2 reviews LFA, its countermeasures, and deep learning including CNN and LSTM. Section 3 describes the system model and problem statement. Section 4 first gives the concept of SCL and then describes its detailed operation. Section 5 demonstrates the experiment results and implications. Finally, conclusions and future works are discussed in Section 6.

## 2. Related Work

This chapter first introduces LFA and related works on defending against LFA. Then, we briefly review deep learning, including CNN and LSTM.

### 2.1. LFA and Countermeasures

**Figure 1:**
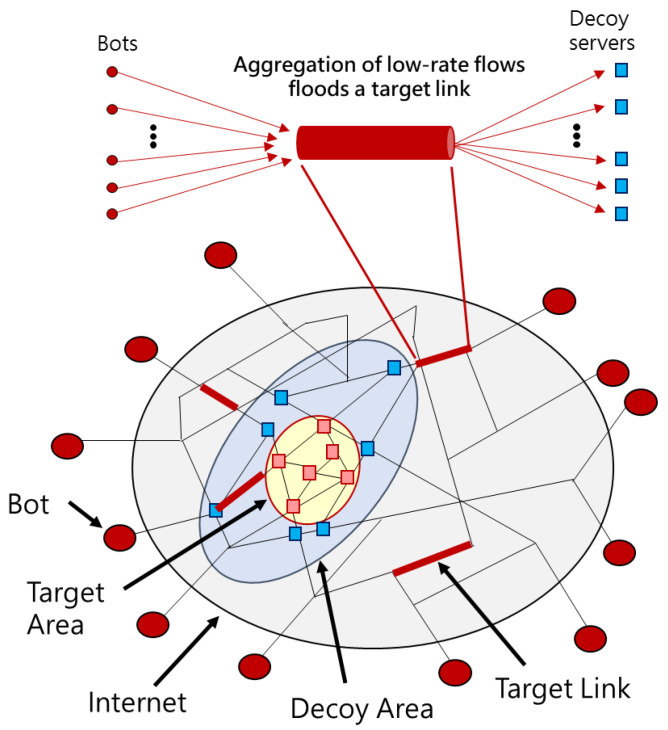
Link flooding attacks (LFA) situation.

DDoS attacks against Internet servers have been around for some time. In contrast, LFA that effectively disconnects selected Internet servers is not common, probably because of the complexity of selective server positioning. This is the biggest difference between DDoS and LFA. The LFA scenario is shown in Figure 1. The red links are target links, which the adversary chooses to flood for the purpose of disconnecting from the Internet. The yellow area is the target area, for which the adversary usually selects the servers of a company, a region, and a country. The target links are those the adversary intends to flood, and the servers in the target area are the real targets that the adversary intents to paralyze. The orange nodes in the target area are public servers, by which the adversary chooses to build an attack topology centered on the target area. The blue nodes around the target area are decoy servers, which are used to create attack traffic. There are some bots at the outer ring, and they are a collection of hacked network devices, which are infected by malware so that the adversary can control them [15].

A general attack procedure of LFA is as follows. LFA first detects the path from the bot to the public server by sending traceroute packets and builds the topology map. Then, the attacker selects some core links in the networks as the target links. Finally, an adversary launches a large-scale, coordinated attack against target links. In order to optimize the attack effects and remain undetected, LFA utilizes numerous sets of target links for the same target area and there are different effects between the sets of target links. LFA uses the best sets of target links most of the time and alternates to the non-best sets of target links merely for a short period of time. For example, if the attacker arranges to attack the best set of target links repeatedly for three minutes, and attack the second-best set of target links in the next 30 s. This attack pattern continues the attack for the target area and shows LFA have a spatiotemporal attack pattern [2].

This section reviews the theses of defending against LFA and organizes them into Table 1. The previous works focusing on detecting LFA can be classified into two types: flow metric threshold [1,3,4,5,6,7,8] and traceroute packet monitoring [9,10]. Because LFA sends large-volume traffic, there will be some variation in traffic rate and consumed bandwidth in links. Therefore, the category of flow metric threshold determines whether LFA happen according to some flow metrics exceeding their corresponding thresholds. The authors of [1] propose an attack detection and mitigation scheme called LFAD in SDN. Since the SDN controller has a globe view, it can easily identify target links by detecting high flow density links. By dynamically deploying a link congestion monitor at each end of the target links to capture traffic data and send them to the SDN controller, LFAD can detect target links whether congested or not. The study in [3] presents SDHoneyNet, an SDN-based system that exposes fake topology to attackers. The paper finds potential bottleneck links by computing the consumed bandwidth rate of each link. Then, SDHoneyNet deploys the honey topology to mimic complex networks. In [4], the authors propose an attack detection scheme, Woodpecker, upgrading several nodes to SDN switches called SDN-enabled nodes. When the packets come into the SDN-enabled node 30 s later, the SDN-enabled node checks the statistical information over the predefined condition threshold to locate the attack. The research study of [5] proposes an attack detection scheme called RADAR based on unmodified commercial off-the-shelf SDN switches. The system monitors the change in the following: flow pattern, link utilization, the number of congestion links and the time of congestion to detect the attack. If these metrics change, then the system determines that the attack has happened. In [6], the authors propose a network defense mechanism, called LinkScope, based on the features when LFA happen such as packet loss rate, round-trip time (RTT), and available bandwidth. The defending system runs on the end host (e.g., the server in a target area) to capture abnormal performance degradation to detect LFA. The study in [7] proposes a randomized security patrol to defend against LFA. By formulating the LFA detection problems to a Stackelberg security game, the paper finds a solution to quantify the attack behavior to detect LFA. In [8], the authors propose an attack detection scheme, BALANCE, heuristically selecting specific nodes to upgrade to hybrid-SDN to get traffic of all links. The system monitors the change of nine metrics, like average link utilization, the standard deviation of bytes sent, and average bytes per packet. If these metrics change, then the system determines that the attack has happened.

The second is to monitor the traceroute packet. To launch effective attacks on target links, the attacker first needs to acquire the topology and usually uses the traceroute packet. Therefore, many traceroute packets will be generated before launching the attacks, so the category of traceroute packet monitoring observes the growth of the traceroute packets. In [9], the authors propose an attack detection scheme that analyzes hop count to the destination to detect LFA. Compared with legitimate users’ destination of traffic distributed evenly, the destination of traceroute, which is caused by the adversary’s reconnoitering topology near the target region before the attack, aggregates within several hops from the target link. The paper therefore periodically observes accessing the target area network traceroute packet, by using hop count to eliminate the malicious traffic before the attack occurs. The study in [10] deploys the SDN controller to monitor ICMP (Internet Control Message Protocol) packets periodically to build the traceroute profile database, due to traceroute using ICMP packets, to identify the potential target links that can be attacked. Furthermore, if the number of traceroute packets is over the threshold, the system considers that there is an attack.

In tradition, detecting LFA can be classified into two types mentioned above. There are two studies that use deep learning methods to detect LFA [11,12]. There are normal traffic and anomalous traffic when the attack happens, so this category of deep learning method classifies two types of traffic by artificial neural networks. In [11], the authors suggest an LFA attack detection scheme for SDN called Cyberpulse that leverages LFA traffic flow statistics to train the ANN module and then classifies them as normal and abnormal flows. The authors of [12] propose an attack detection scheme for SDN called LF-Shield that also utilizes LFA traffic statistics to train the CNN module and then classifies them as normal and abnormal flows.

The previous work focusing on mitigating LFA can be classified into two types: traffic rerouting [1,10] and blacklist [4,5,8,11,12,13]. When LFA occurs, the target links are congested continuously. Therefore, the category of traffic rerouting reroutes flows which originally travel through the target links to relieve their congestions. In [1], the authors use a multiple path rerouting approach to mitigate the effect of LFA congesting many target links at the same time. The approach chooses several optional paths and determines how much traffic should be routed to the optional links. After rerouting, the attacker needs to find a new target link. In this way, the LFA bots can be found, and then the malicious traffic is dropped from the LFA bots. The study in [3] deploys moving target defense (MTD) on the SDN controller. Once the suspicious attack is found, the system sets rules of the SDN switches to randomize the routes for ICMP packets, which mutates the routing path to make it difficult for the adversary to launch the attacks. Simultaneously, when the system detects the congested link, the route mutation mechanism will also be activated.

The category of blacklist involves collecting suspicious flows into a blacklist and drop packets based on this blacklist when the LFA have been detected. In [4], once the LFA are detected, the centralized traffic engineering is activated. The traffic engineer broadcasts the “block route message” to all routers. In [5], the authors identify flow as abnormal, if flow rates of statistic changes correspond to those of aggregated flows delivered on victim links. Then, the system drops the traffic based on a blacklist. To avoid falsely dropping the normal traffic, the system uses max-min fairness packet dropping, which is when most packets matching the features of the attack traffic are suspected and can be regarded as the attack traffic if the number exceeds the throttle at each OpenFlow port. The study in [8] puts suspicious source IP into the bot blacklist, randomly picks 20% of bots from the blacklist, and discards the packets from them. After classifying benign and malicious traffic, the authors in [11] forward the consequence to the flood light controller. The controller uses the null routing method to mitigate LFA, which means if the packets match a null route, it will be discarded. The authors in [12] drop packets based on the blacklist, and use a max-min fair bandwidth-limiting mechanism to limit the bandwidths. The study in [13] manages the throughput via the tail drop technique, which means if the aggregate flow rate reaches capacity, the gateway buffers the excess data in a queue, waiting to be transmitted. When the queue is filled to its maximum capacity, the newly arriving flows will be dropped until there is enough room to accept incoming flows and if the network is congested, fewer packets will be sent per RTT.

Numerous artificial intelligence (AI) methodologies [11,12,14,15,16,17,18,19,20,21,22,23,24,25] are introduced to defend against LFA without any manual intervention [26,27,28,29,30]. The basic idea of the current AI-based methodology is to screenshot the current network status as the input sample, then to apply convolution and pooling operations to extract features of the LFA, and finally to decide the possibility of the current network suffering LFA. However, these AI-based methods adopt a simple deep learning architecture that can only focus on LFA detection rather than simultaneously coupling with actions of detecting and mediating the LFA. On the other hand, they only use one network status at a certain time slice and neglect the fact that LFA are a continuous attacking action or combination of varying attacking actions, since LFA are gradually generated, enhanced, and dynamically switched. 

**Table 1:** LFA detection and mitigation comparison table.

No.	SDN	Detection				Mitigation	
		Detect Target/All Links	Flow/Packet Based	Method	Time Series	Method	Related to * of TLs
[1]	Y	Target	Flow	Measure TL U	N	1.Rerouting 2.Drop malicious flows	N
[3]	Y	All	Flow	Consumed Bandwidth Rate > T	N	NA	N
[4]	Y	All	Flow	Traffic rate > T	N	Broadcast block route message to all routers	N
[5]	Y	All	Flow	Rate of link U changes > T	N	1.Max-min fairness packet dropping 2.Drop flows based BL	N
[6]	N	All	Flow	Available Bandwidth > T	N	NA	N
[7]	N	All	Packet	Randomized Traffic rate > T	N	NA	N
[8]	Y	All	Packet	Traffic rate > T	N	Random dropping	N
[9]	N	All	Packet	Monitor the traceroute packet	N	NA	N
[10]	Y	All	Packet	Monitor the traceroute packet	N	MTD	N
[11]	Y	All	Flow	ANN	N	Null routing	N
[12]	Y	All	Flow	CNN	N	1.Drop packets based BL 2. Max-min fairness packet dropping	N
[13]	N	NA	NA	NA	N	Tail drop	N
[26, 27, 28, 29, 30]	Y	All	Flow	Stacking based Deep Learning method	Y	NA	N

* TL: target link, U: utilization, T: threshold, MTD: moving target defense, BL: blacklist, TE: traffic engineering, ANN: artificial neural network.

### 2.2. Deep Learning

Deep learning has been widely applied in many domains including predicting passenger volume for urban rail systems [34], Cardiac Arrhythmia disease classification [35], and malware analysis [36,37]. The study in [34] applies a long short-term memory model (LSTM) to forecast short-term outbound passenger volume at urban rail stations. In [35], the authors adjust LSTM by appending the functionalities of the Principal Components Analysis and full-connected layers to classify the Cardiac Arrhythmia Disease in order to solve the overfitting problem. Two other studies [36,37] both apply LSTM to analyze the sequence of machine codes in order to detect the malware.

This section briefly introduces the core concept of two DNN models, CNN and LSTM used in this study [16,17,18,19,20,21,22,23,24,25].

#### 2.2.1. CNN

**Figure 2:**
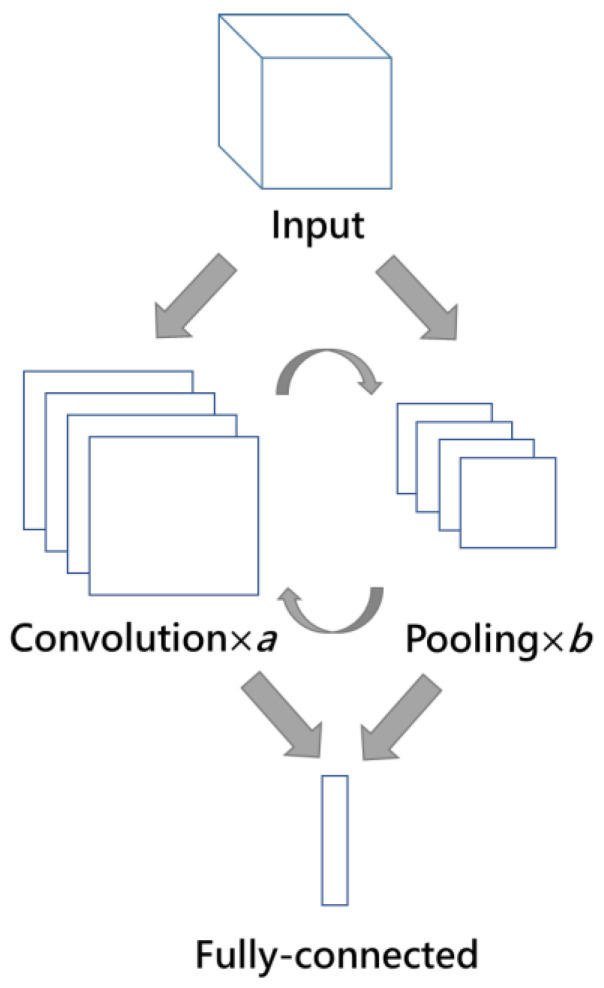
Convolutional neural network (CNN) architecture.

CNN is usually used in image processing which includes detecting objects, classifying images, and recognizing objects by performing feature extraction and mapping through fast training. Rather than analyzing a whole image at once to find certain features, CNN can be more effective to look at smaller parts of the image and has a high prediction accuracy. The main purpose of CNN is to reduce the images into a form that is easier to operate, without losing features that are critical for getting a good prediction. CNN mainly includes three layers, namely the convolution layer, pooling layer, and fully connected layer. Figure 2 shows the architecture of CNN where the final step is the fully connected layer. The order of convolution and pooling layers and the number of convolution and pooling layers are decided by the designer, which means there are many combinations of CNN modules. The input of CNN could be two-dimensional, three-dimensional, and four-dimensional, and inputs are data. In the convolutional layer, the input is processed by a moving function called a filter, which selects the feature in the image. In the pooling layer, the input is sampled to a smaller one. Then, the fully connected layer classifies the output into one category [16]. The elements of CNN are described as follows.

A. Convolutional layer

The convolutional layer acts as a kernel role of the CNN and the objective of the layer is to extract high-level features, such as edges, from the input. The parameters of the convolutional layer comprise learnable filters. These filters pass through the full input area by picking up a small region of the input at a time. The filter values are multiplied with the corresponding pixel values and the sum of the products is computed as the output for that region. After the filter traverses the whole image space, one feature map will be generated and then the results are passed into a nonlinear activation function. The length of the filter movement step, called stride, can be adjusted by the designer. There are many commonly used activation functions, such as sigmoid, rectified linear unit (ReLU), and hyperbolic tangent function (tanh). If several filters are used, several feature maps are obtained, which compose the output of the convolutional layer. Since the full input uses the same filter at each time, through sharing the weight of characteristics in the filter which generates a feature map, the convolutional layer can extract the characteristics of input and can use fewer parameters to reduce the model’s complexity [17].

B. Pooling layer

The aim of the pooling layer is to reduce the dimensions and increase the robustness of feature extraction for decreasing the computational power required on parsing the data. The typical pooling operations are average pooling and max pooling, which are designed to pick up the average value or maximum value, respectively, from the region covered by the filter, which, as with the convolutional layer, is a small region traverse through the entire image area. Likewise, the length of the filter movement step, called stride, can be adjusted by the designer. Max pooling can play the role of de-noising while average pooling is only for dimension reduction. Therefore, in general, the effect of max pooling is better than that of average pooling [18,19].

C. Fully Connected layer

The purpose of the fully connected layer is to take the output of the convolutional layer or pooling layer and use them to output a one-dimensional array. The array contains the probability of each label option. The total probability of all options is one. This probability is used to provide a basis for selection. The results of convolution/pooling layers are flattened into a one-dimensional vector of values where each value represents a probability that a certain feature pertains to a label. One or more fully connected layers are appended at the end of the CNN which takes all neurons from the previous layer and connects it to all neurons in the current layer. Each neuron obtains weights that prioritizes the most suitable label [20].

#### 2.2.2. LSTM

**Figure 3:**
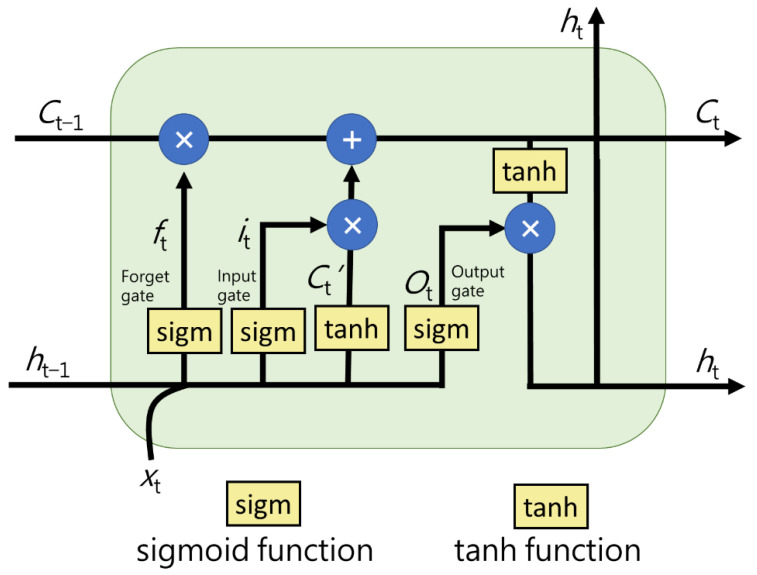
Long short-term memory (LSTM) architecture.

LSTM is a model of DNN and evolved from the Recurrent Neural Network (RNN). In contrast to other DNN architectures, the inputs and outputs are one fixed vector; therefore, RNN is able to input and output sequences of vectors, which is why RNN is adept at solving time series problems. The reason to develop LSTM is that LSTM has the superiority of conquering the vanishing and exploding gradient problems that RNN suffers from. This is because LSTM improves RNN with memory cells, a stored unit, simplifying the learning of temporal relationships over long time scales [21]. LSTM has a function of removing and adding information from a memory cell, which is controlled by structures called gates. The role of gates is to decide how much of the overall proportion of information to let through. Gates are composed of a sigmoid neural net layer and a pointwise multiplication operation. By outputting value between zero to one, the sigmoid neural net layer regulates the degree of information that should be let through in each component. If the value of the sigmoid neural net layer is zero, it means let nothing through. If the value of the sigmoid neural net layer is one, it means let everything through. As shown in Figure 3, the green square is a network, and LSTM has multiple flows of this network. LSTM commonly consists of a memory cell, forget gate, input gate, and output gate to control how to update the value by contrasting the inner memory when new information arrives.

A. Memory cell

A memory cell is a key to LSTM which stores a computing value (or state) for a defined period. It is the horizontal line running through the top of Figure 3. Removing and adding information from a memory cell is regulated by gates.

B. Forget gate

The forget gate regulates the proportion of an input value that stays in the memory cell, which means to determine what information we are going to throw away from the memory cell. Forget gate activation function is a sigmoid layer. In Figure 3, input *X*_t_, which is also the output of the last cycle, goes through a sigmoid layer and outputs a number between zero to one for every number in the memory cell *C*_t−1_. A value of zero stands for totally remember the number while a value of one stands for totally forgetting the number.

C. Input gate

The input gate regulates the proportion of an input value that is allowed to flow into the memory cell, which means to determine what new information we are going to store in the memory cell. As illustrated in Figure 3, there are two parts in the input gate. First, a sigmoid layer called the input gate layer determines which values LSTM will update. Next, a tanh layer creates a vector of new candidate values, *C*_t_’, that could be added to the memory cell. Then, we will combine these two values to create an updated value to the memory cell. Third, LSTM will update the old memory cell, *C*_t−1_, into the new memory cell *C*_t_. We multiply the old state by *f*_t_, forgetting the things we decided to forget earlier in the forget gate, and then we add *i*_t_ × *C*_t−1_. These are the new candidate values, scaled by the degree to which we decided to update every state value.

D. Output gate

The Output gate regulates how much of the value in the cell is used to calculate the output of the LSTM unit, which means to determine what we are going to output, as illustrated in Figure 2. First, we run a sigmoid layer which determines what parts of the memory cell we are going to output. Then, we put the memory cell through tanh (to push the values to be between minus one and one) and multiply the value by the output of the sigmoid gate, *O*_t_, and then output *h*_t_. Therefore, LSTM only outputs the parts we decided to output.

## 3. Problem Statement

**Figure 4:**
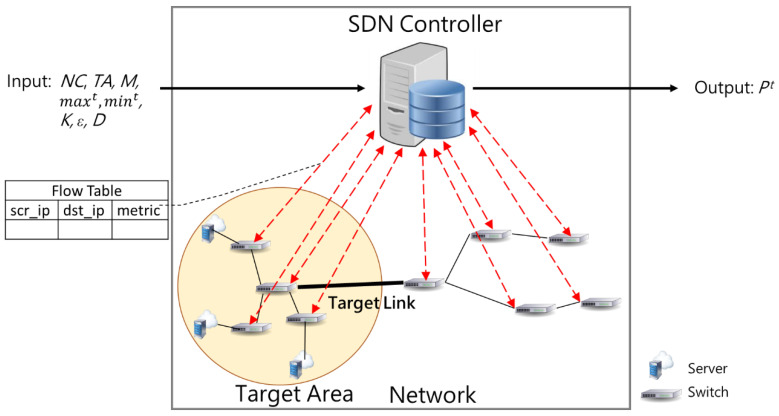
System model.

Table 2 summarizes the notations used in this study and their corresponding meanings while Figure 4 illustrates the overall system model architecture. Assume the input of SCL is composed of *z* nodes. Therefore, the dimension of network capacity matric, *C*, is *z* × *z*, where the element *c_i_*_,*j*_ is the link capacity from node *i* to *j.* The network traffic matric at time *t*, denoted as *S^t^*, also has the dimeson of *z* × *z*, where the element *s_i_*_,*j*_ is the amount of traffic from node *i* to *j* at time *t.* SDN controller gets the total data of the Internet from the switch. We run SCL in the SDN controller after getting the data of switch and then we send computing results to the switch.

First, the system gets the information of *NC*, *NS^t^*, and *TA*, and then SCL detects whether LFA happen and the target area is given; all links that get into the target area are target links. If SCL detects that LFA happen, SCL will determine dropping probabilities in some links and drop some packets according to these values to mitigate the congestions in these links. Finally, the output of our problem is dropping probabilities in each target link. Our objective is to maximize *M*^A^, which is the accuracy of deciding whether the system should drop packets or not. Therefore, the problem statement is defined as below.

**Given:***C*, *TA*, *M*, *max^t^*, *min^t^*, *K*, *ε*, and *D*
**Output:** dropping probabilities in target links at time *t*: *P^t^*
**Objective:** maximize *MA^t^*

## 4. Stacking-Based CNN and STM(SCL)

In this chapter, we first show the concept and the overall architecture of SCL, and then explain the embedded modules in detail.

### 4.1. SCL Overall Architecture

SCL stacks two DNN models, CNN and LSTM, to defend against LFA. SCL can learn how to detect LFA automatically and alleviate attacks according to the ratio of attacked target links. SCL exploits the CNN model because it can extract features and reduce dimensions, so it can be trained in a short time. SCL uses the LSTM model, which has a time–space concept, so it is very suitable for detecting LFA, which has a spatiotemporal attack pattern. Finally, SCL mitigates LFA based on the ratio of attacked target links because the number of attacked target links is related to the seriousness under attack.

**Figure 5:**
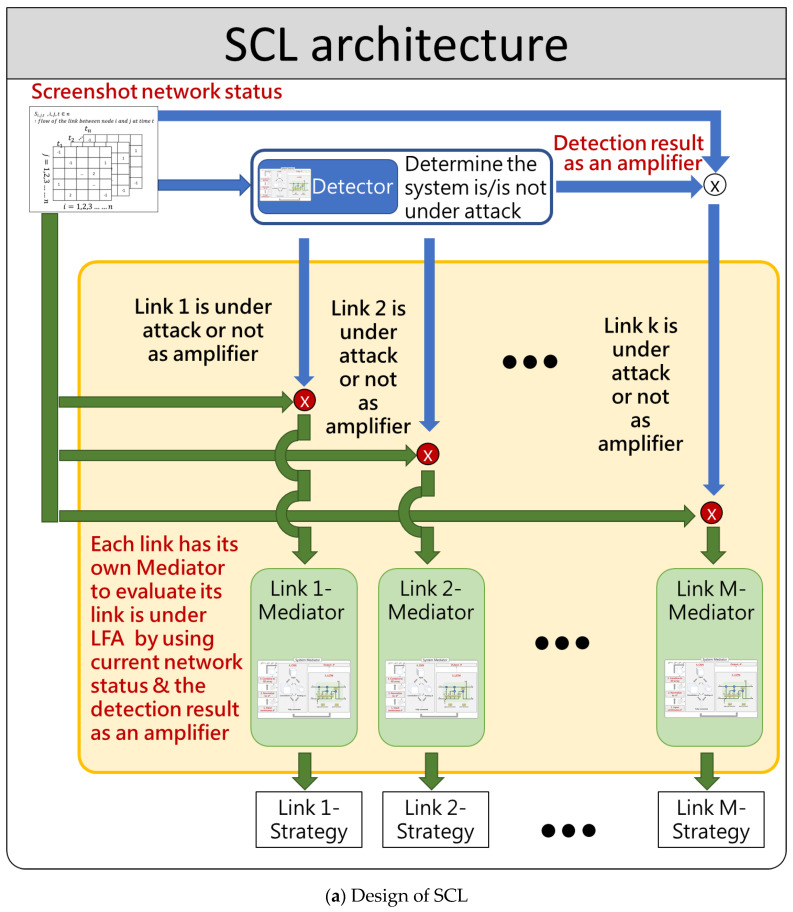

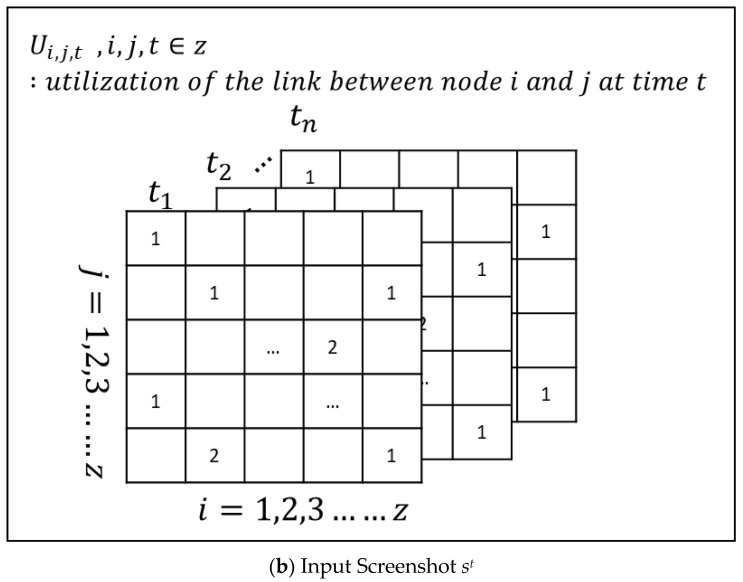
Architecture of CNN-LSTM (SCL).

The whole SCL framework is shown in Figure 5a. SCL applies ensemble learning architecture which consists of two AI layers: the first layer is the System Detector module and the second one is the LFA Mitigator module. System Detector is responsible for detecting if the system is under attack and the LFA Mitigator module is designed to mitigate the LFA when the module determines the attack has reached the level to be mitigated. Both two modules are composed of CNN and LSTM algorithms. Once the LFA Mitigator module finds the network incident happening, the LFA Mitigator module will locate the attack and heuristically calculate the packet dropping rate according to the seriousness of the attack after determining that the attack has upgraded to network attack from network incident. The strengths and the novelty of SCL in adopting two layers of AI modules can obtain better predictive performance than could be obtained from any of the constituent learning algorithms alone. Just like the military operations, the System Detector plays a role of scout to explore an area to gain information about network flow features. Once the System Detector detects an unusual flow, it then raises the alarm by amplifying the network flow features. Then, each link uses its own mediator to evaluate its link is under LFA by using current network status and the detection result as an amplifier.

Both System Detector module and LFA Mitigator module use deep learning, that is, they are composed of CNN and LSTM. The reason why both of these two modules use CNN is because the parameters in the sample are still very large and cannot converge. Therefore, CNN is needed to remove unnecessary noise data and reduce the dimensions to increase the operation speed in the following step. System Detector utilizes LSTM because when LFA launch an attack in a short time, there are a lot of periods during which the link utilization is normal. Therefore, SCL needs LSTM, which has a time series concept, to predict which period is most likely for the attack to happen based on the flow pattern. On the other hand, the reason why LFA Mitigator module uses LSTM is that LFA dynamically change a subset of target links to attack, meaning that the attack keeps taking turns to attack different target links in different periods. Therefore, we utilize historic time-order data, as shown in Figure 5b, to predict the next period in which the attacker will attack and the corresponding set of target links, which is very suitable for LSTM.

### 4.2. System Detector Module

**Figure 6:**
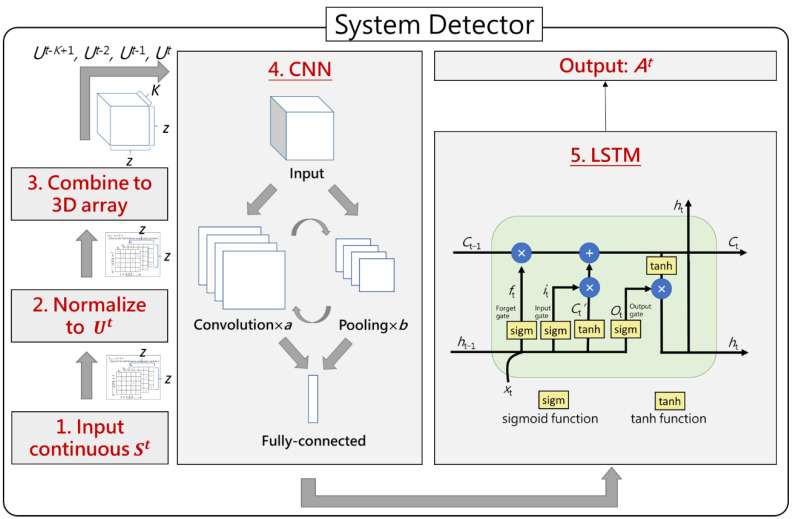
Architecture of System Detector module.

System Detector module inputs St, which is traffic status at time t, and the final output is At, which is system is under attack or not. The architecture of System Detector module is shown in Figure 6. First, System Detector module screenshots each time of link traffic St, and normalizes St to Ut through $s_{z,z}^{t\;}$, each element in St, divided by $c_{z,z}$, each element in C, to calculate $u_{z,z}^{t\;}$, each element in Ut. Then, in the third step, System Detector module combines consecutive K sheets of Ut into a three-dimensional array according to time, which is $u_{z,z,K}^{t\;}$, and it is a sample of combination LFA for the following fourth step of CNN. The reason for the third step is that combining consecutive input as a three-dimensional array makes the input a chronological relationship. Therefore, SCL can predict the flow pattern according to the time sequence. The fourth step is CNN, which is composed of convolution, pooling, and fully connected layers, and we use ReLU as our activation function in CNN; the reason why we use ReLU as our activation function will be explained in Section 5.2.4. After convolution and pooling layers remove unnecessary noise and reduce the array dimensions, the fully connected layer flattens the sample into a one-dimensional array; the array contains the probability of each label option, and the array is the input of the following fifth step of LSTM. Finally, when the gap of DA (Detection Accuracy of system under attack) with last time is less than a specific value *ε* for D times, the training of System Detector module ends.

The input sequence of CNN is *U^t^*^-*K*+1^, *U^t^*^−2^, *U^t^*^−1^, *U^t^* at the first time, and the input sequence of the next time is *U^t^*^-K+2^, *U^t^*^−1^, *U^t^*, *U^t^*^+1^. That means SCL removes the first position, *U^t^*^-*K*+1^, adds the new one, *U^t^*^+1^, and the second, third, and fourth positions overlap with the previous time. For example, if CNN inputs the first, second, third, and fourth second of *U^t^* for the first time, the input of next time will be the second, third, fourth, and fifth second of *U^t^*. As for output, when CNN input *U^t^*^-*K*+1^, *U^t^*^−2^, *U^t^*^−1^, *U^t^*, the output will be *A^t^*. That is, the input of continual four seconds of network traffic changing plot corresponds to the output of the last second that the action decision should be taken in. For example, if CNN inputs the first, second, third and fourth seconds, the output will be the prediction of system under attack or not of the fourth second.

### 4.3. LFA Mitigator Module

**Figure 7:**
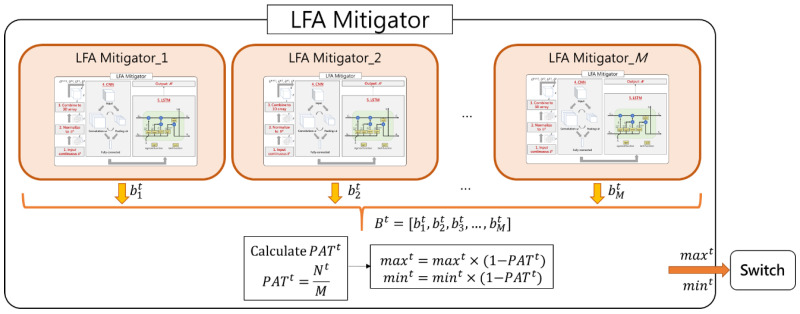
Architecture of LFA Mitigator module.

LFA Mitigator module inputs *S^t^*, and the final output is *P^t^* which denotes the dropping probability of each target link. The architecture of LFA Mitigator module is shown in Figure 7. In mitigating LFA, we must first know where the target links are. In SCL, similar to the previous work, we use a simple approach to select the border links in the target area as target links. Note there are *M* LFA Mitigator modules where *M* is the number of target links because one LFA Mitigator module exists for each target link. The main reason is that each target link has two prediction results, attack or not attack. In the SCL method, it is more accurate to predict by single-choice questions, which is *M* LFA Mitigator modules and each of them outputs one bit. If SCL predicts by multiple-choice questions, which is only one LFA Mitigator module, and outputs eight bits, it will be not that accurate. Then the *i*-th LFA Mitigator outputs one bit to indicate whether its corresponding target link is under attack or not. That is, after the deep learning methods are finished, a vector $B^{t}=\left[b_{1}^{t},b_{2}^{t},b_{3}^{t},\ldots ,b_{M}^{t}\right]$ is obtained. Finally, LFA Mitigator obtains this vector and understands the seriousness of the attack, and calculates the vector $P^{t}$ which denotes the dropping probability of each target link.

Consequently, the more target links are under attack, the more serious the attack is, and, thus, the higher the probability of dropping the packets. Therefore, LFA Mitigator module integrates information of whether all target links are under attack or not to calculate the probability of attacked target links, $PAT^{t}$, which is the number of attacked target links, $N^{t}$, divided by the number of target links, *M*. We design a mechanism to determine the probability of packets dropping, $P^{t}$, based on the $max^{t}$ and $min^{t}$, which are maximum and minimum thresholds of link utilization at time *t*. The lower $max^{t}$ and $min^{t}$ are, the earlier the system begins to drop packets, which means less link utilization. Consequently, $max^{t}$ and $min^{t}$ are multiplied by $\left(1-PAT^{t}\right)$ and then new thresholds are sent to switch in the target links. The switch in each target link will compute $P^{t}$ based on $max^{t}$ and $min^{t}$. $Max^{t}$ and $min^{t}$ and $P^{t}$ are linear relationships, which means $max^{t}$ corresponds to $P^{t}$ equal to one and $max^{t}$ corresponds to $P^{t}$ equal to zero. When the link utilization exceeds $max^{t}$ the system drops all packets and when the link utilization is below $min^{t}$, the system does not drop packets. Moreover, when the utilization is between $max^{t}$ and $min^{t}$, the $P^{t}$ is calculated based on linear relationship. The formula of $P^{t}$ is given in Equation (1).

(1) $$P^{t}=\frac{U_{z,z}^{t\;}-max^{t}}{max^{t}-min^{t}},\;P^{t}\;\left[0,1\right]$$

## 5. Evaluation

The detailed environment settings and the experiment results are demonstrated in the following section.

### 5.1. Scenario and Parameter Setting

In this section, we will explain how to set up the scenarios and parameters, all experiment scenarios, evaluate the detection performance metrics, and the methods to be compared.

#### 5.1.1. Scenarios Setting

**Figure 8:**
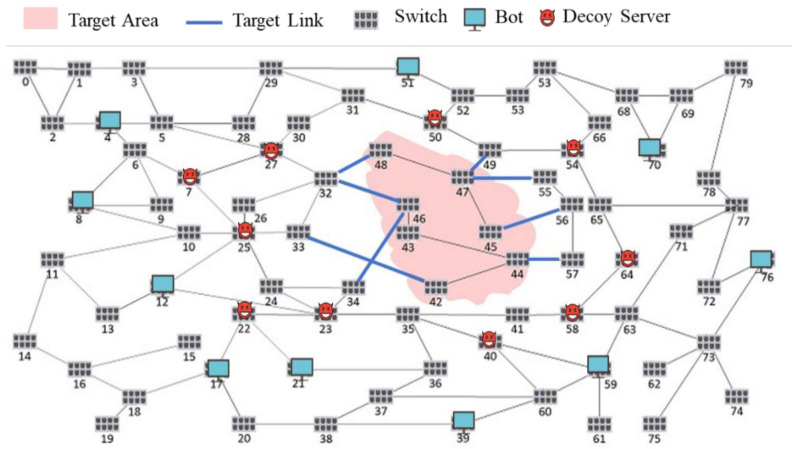
Experiment topology.

The topology of this experiment contains 80 switches and varying devices including bots as shown in Figure 8. The pink part on the graph is the target area we are given, which indicates that the devices in this area shall be protected. The attack scenario parameter settings are shown in Table 3. All links that get into the target area are target links that are shown in blue lines of link in Figure 8. Each link capacity is 60 Mbps, and the routing method we use is the shortest path. As for normal traffic, we generate 1 to 50 flows each time, and source and destination are random nodes in the topology. The flow traffic is 1 to 60 Mbps, the time of each normal traffic is randomly decided, and we launch normal traffic every one second. 

Considering that the current learning/testing dataset has an imbalance in class distribution, the performance and the comparison between methods will be miss-interpreted. For example, if 99% of samples in a dataset belong to one class, the testing model will always achieve an accuracy of 99%. This study randomly generates LFA attacking and normal traffic (50% vs. 50%) based on a fuzzy testing methodology to ensure all experiments in this section have sufficient samples/scenarios regarding the LFA attacking and normal traffic for training and testing. There are 10 bots randomly dispersed outside the pink area. All bots randomly launch attacks by sending flows that go through the blue links, which are the target links, to get into the pink area and go out of the pink area which is in order to paralyze the service in the target area. Each bot randomly generates multiple flows that are capable of paralyzing the target links, and each flow contains 4 kbps of attack traffic.

#### 5.1.2. SCL Parameter Setting

The details of System Detector module and LFA Mitigator module and CNN parameter setting are demonstrated in Table 4 and Table 5. In this case study, the two-dimensional network capacity of C is five hops away from target links (75 × 75) and K is four, which means every continuous four Ut are combined as the input of CNN. Therefore, the dimension of CNN input is 75 × 75 × 4. The final output of CNN is a one-dimensional array (1 × 512). In this experiment of CNN part, SCL adopts three convolution layers and one pooling layer. The reason for setting parameters in the above will be explained in Section 5.2 and Section 5.3.

#### 5.1.3. Performance Evaluation Metric

We use four metrics for performance evaluation, they are detection accuracy (*DA^t^*), mitigation accuracy (*MA^t^*), overall training time (*OTT*), the relationship between *DA^t^* and *OTT* (*A2O*), FRR (False Acceptance Rate), and FAR (False Acceptance Rate). First, *DA^t^* is detection accuracy, which is used to evaluate System Detector, and its calculation method is given in Equation (2). Second, *MA^t^* is mitigation accuracy, which is used to evaluate LFA Mitigator, and its calculation method is given in Equation (3).

(2) $$DA^{t}=\frac{DTP^{t}+DTN^{t}}{DTP^{t}+DFP^{t}+DFN^{t}+DTN^{t}}$$

(3) $$MA^{t}=\frac{MTP^{t}+MTN^{t}}{MTP^{t}+MFP^{t}+MFN^{t}+MTN^{t}}$$

Third, *OTT* is the measure of the overall training time in the deep learning method, which is a factor of evaluating the best deep learning parameter in Section 5.2 architecture investigation. The System Detector ends when the gap of *DA^t^* with last time is less than 0.2% for 15 times, so that *DA^t^* corresponds to the number of training times, *NT*; we calculate how many hours of deep learning is spent in each training time, which is *ET*. *OTT* is the value of *NT* multiplied by *ET*. The formula of *OTT* is given in Equation (4).

(4) OTT=NT×ET

Fourth, *A2O* is the measure of the relationship between *DA^t^* and *OTT* to select the best deep learning parameter in Section 5.2 architecture investigation. The formula of *A2O* is given in Equation (5). *A2O* is the value of *DA^t^* squared divided by *OTT*. Because *DA^t^* is the accuracy of determining if system under attack or not, which is the most important factor to determine how accurately to drop packets in LFA Mitigator, therefore, *DA^t^* will be squared to determine the best parameter for deep learning architecture investigation. *OTT* is the overall training time of a deep learning system, which is also an important factor to determine the efficiency of the deep learning method. Furthermore, the more the *OTT* is, the more cost the system spends. Therefore, *DA^t^* squared is divided by *OTT*.

(5) $$A2O={\left(DA^{t}\right)}^{2}/OTT.\;$$

Finally, FRR and FAR are used to evaluate System Detector, and their calculation methods are given in Equations (6) and (7).

(6) $$FRR=\frac{DFN^{t}}{DTP^{t}+DFN^{t}}$$

(7) $$FAR=\frac{DFP^{t}}{DFP^{t}+DTN^{t}}$$

### 5.2. Architecture Investigation

In this section, we will explain how to set up the architecture of CNN, the experiment of the number of convolution layers, the number of pooling layers, the order of convolution layers and pooling layers, and the activation function to be compared are described.

#### 5.2.1. The Effects of the Number of Convolution Layers

**Figure 9:**
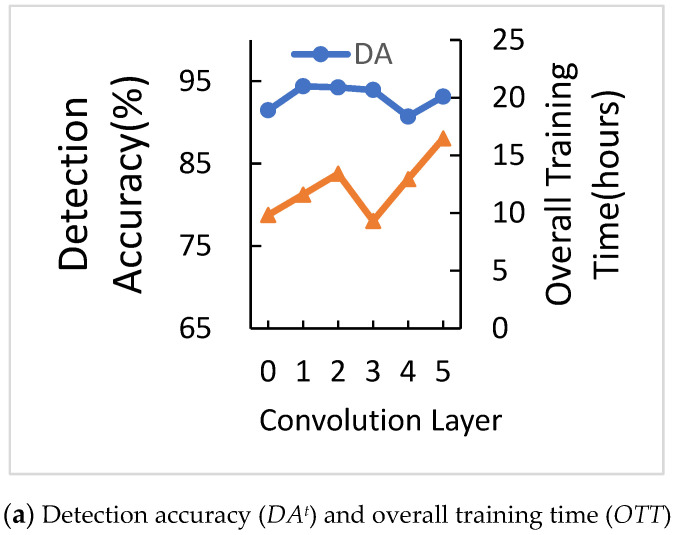

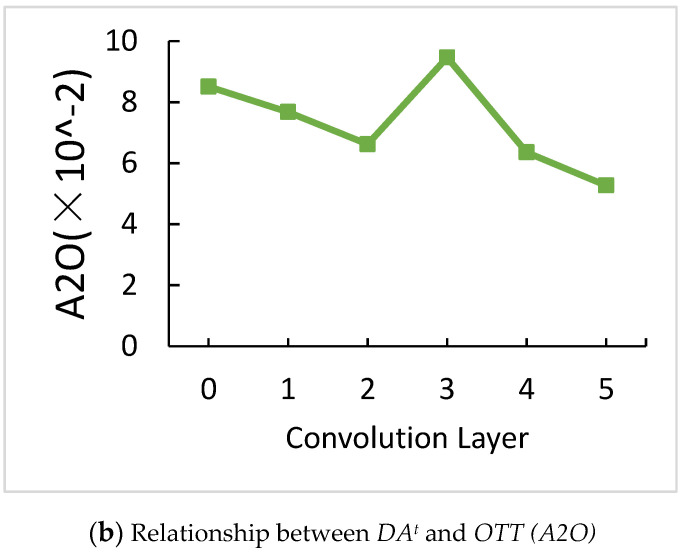
Comparison of the number of convolution layers.

The system ends when the gap of *DA^t^* with last time is less than 0.2% for 15 times. We calculate the average of the last five times, which are (*DA^t^*^−4^, *DA^t^*^−3^, *DA^t^*^−2^, *DA^t^*^−1^, *DA^t^*), as the final *DA^t^*. Figure 9a shows the comparison of the number of convolution layers for *DA^t^* and *OTT*. When the number of convolution layers is ranging from zero to five, *DA^t^* and *OTT* are irregular. The reason is deep learning has its way of training. 

*DA^t^* of one convolution layers is 94.38%, which is the highest, but *OTT* of one convolution layer is not the lowest. Thus, we need a comparison of *A2O* to choose the best parameter. Figure 9b shows the comparison of the number of convolution layers for *A2O*. When convolution layers equal to three, *A2O* is higher than any other number of convolution layers, which shows that three convolution layers have better performance. Therefore, we chose three layers of convolution.

**Figure 10:**
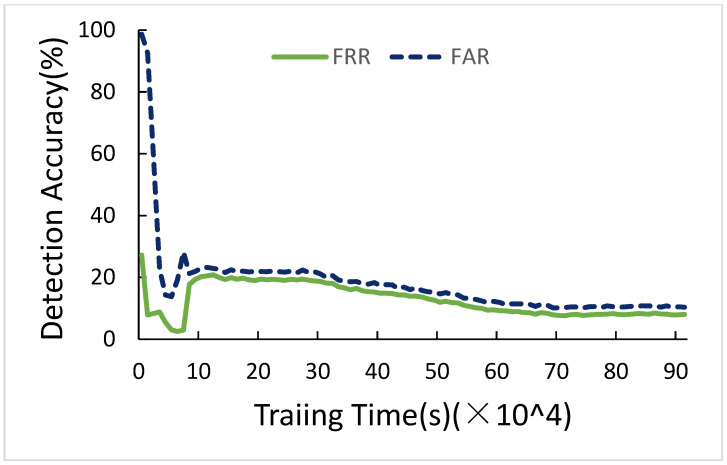
FRR and FAR of three convolution layers.

Figure 10 is FRR and FAR of selected parameters for three Convolution layers. As time goes by, FRR and FAR become lower and lower, and finally, they are 8.01% and 2.20%, respectively. The other sections do not show this figure again, since the results are the same. 

#### 5.2.2. The Effects of the Number of Pooling Layers

**Figure 11:**
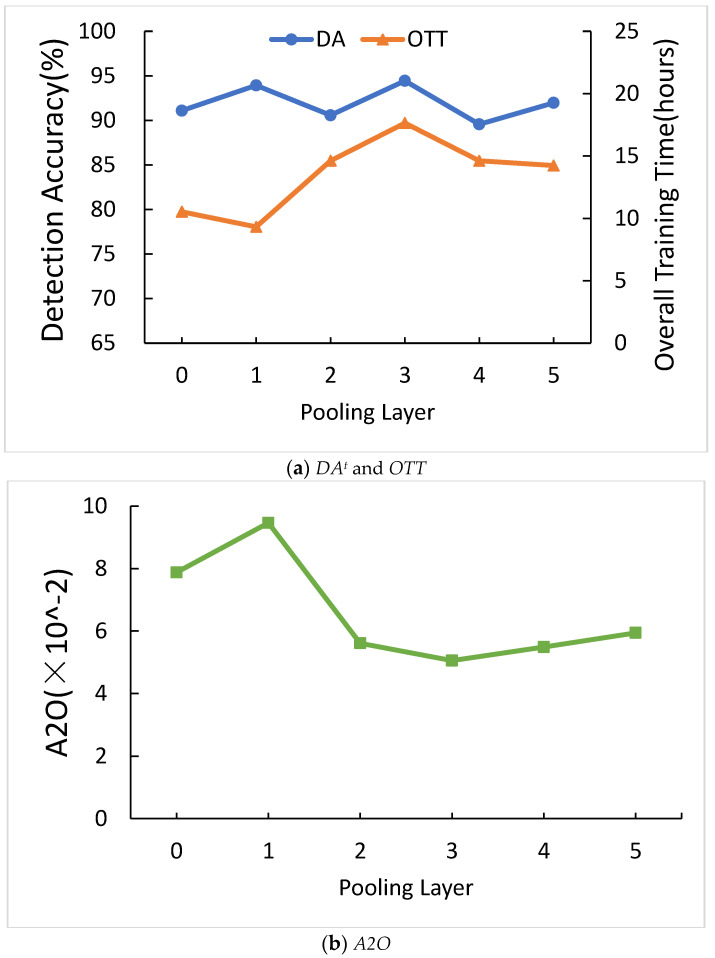
Comparison of the number of pooling layers.

Figure 11a shows the comparison of the number of pooling layers for *DA^t^* and *OTT*. When the number of pooling layers is ranging from zero to five, *DA^t^* and *OTT* are irregular. Figure 11b shows the comparison of the number of pooling layers for *A2O*. When pooling layers equal to one, *A2O* is higher than any other number of pooling layers. One layer of pooling is the highest *A2O*, so we chose one layer of pooling.

#### 5.2.3. The Effects of Different Orders of Pooling in CNN

**Figure 12:**
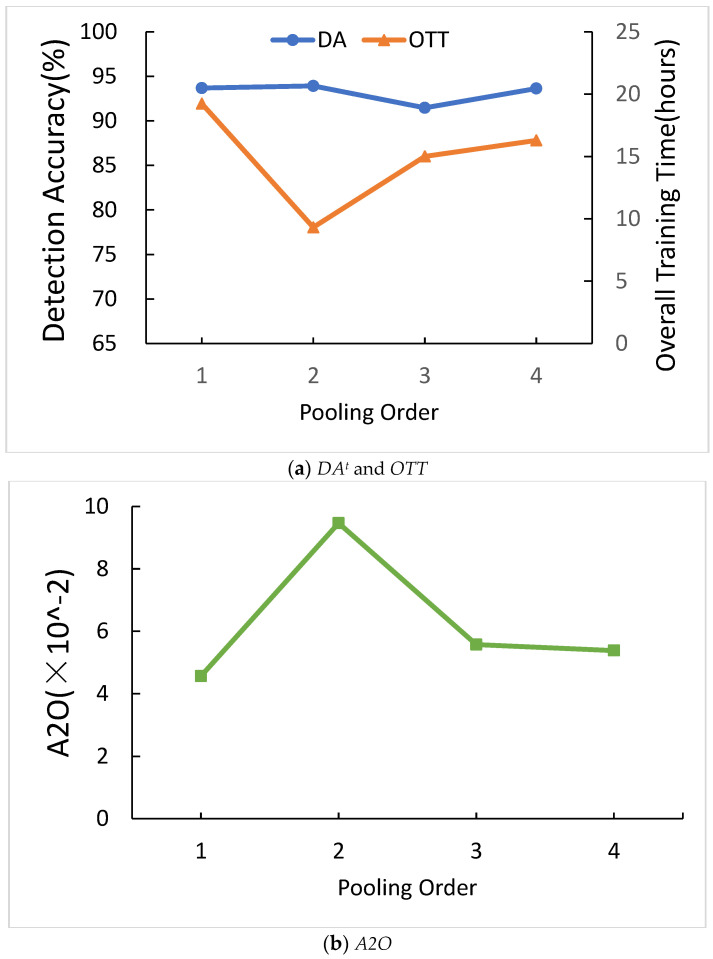
Comparison of different orders of pooling in CNN.

Figure 12a shows the comparison of different orders of pooling in CNN for *DA^t^* and *OTT*. The four types of orders are shown in the below. In the order type of pooling in four types, *DA^t^* and *OTT* is irregular.

(1) Pooling -> Convolution -> Convolution -> Convolution -> Fully Connected;
(2) Convolution -> Pooling -> Convolution -> Convolution -> Fully Connected;
(3) Convolution -> Convolution -> Pooling -> Convolution -> Fully Connected;
(4) Convolution -> Convolution -> Convolution -> Pooling -> Fully Connected.

Figure 12b shows the comparison of different orders of pooling in CNN for *A2O*. When the order of type is the second type, *A2O* is higher than any other order type. Since the second type of pooling order is the highest *A2O*, we chose the second type of pooling order.

#### 5.2.4. The Effects of Different Activation Functions in CNN

**Figure 13:**
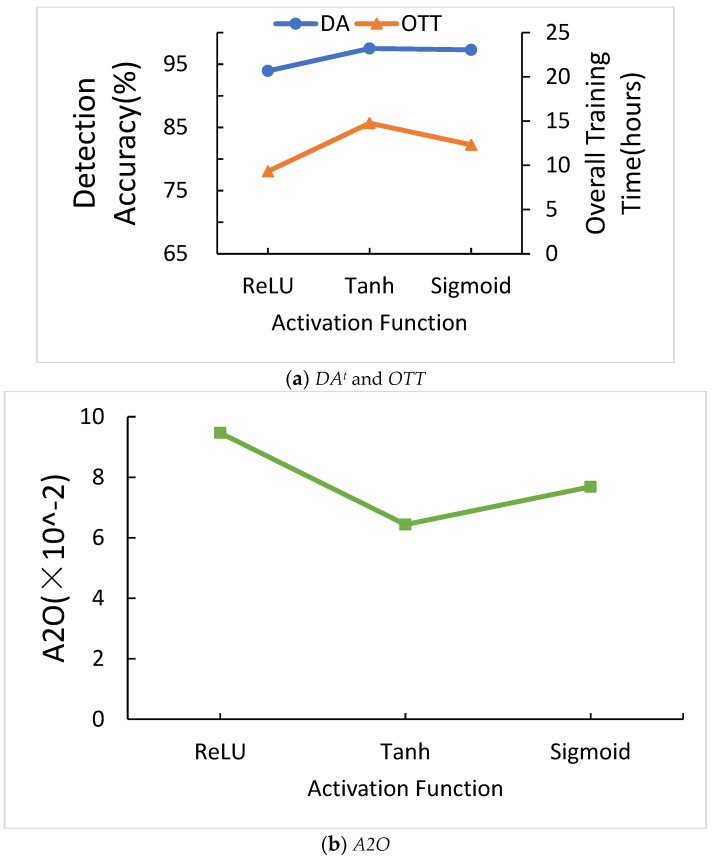
Comparison of different activation functions in CNN.

Figure 13a shows the comparison of different activation functions in CNN for *DA^t^* and *OTT*. When the activation function is ReLU, *DA^t^* is the highest and *OTT* is irregular. Figure 13b shows the comparison of different activation functions in CNN for *A2O*. When the activation function is ReLU, *A2O* is the highest. Thus, we chose ReLU as our activation function. The reason is when x is smaller than zero, all the values of y are zero in ReLU function. Therefore, ReLU function can remove the negative value, and can more extract the feature of the object.

#### 5.2.5. Performance of SCL

**Figure 14:**
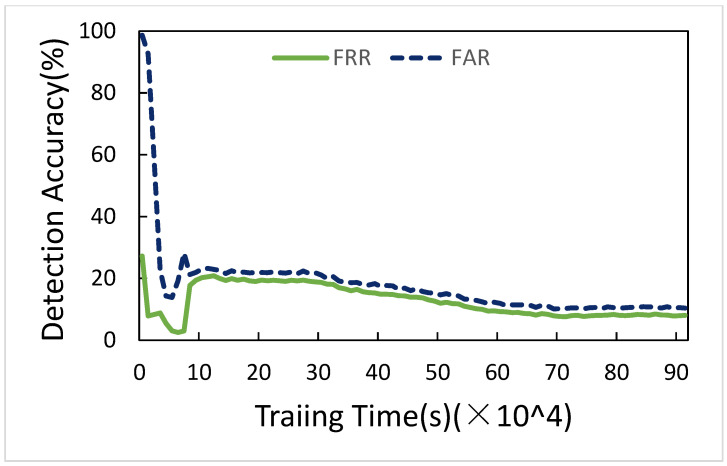
Performance of SCL.

Figure 14 shows the comparison of training and testing. Section 5.2.1, Section 5.2.2, Section 5.2.3, Section 5.2.4 are performances of training. As time goes by, training and testing become higher and higher. The *DA^t^* of training starts from relatively low (52.92%), and the *DA^t^* of testing starts from relatively high (92.43%). Finally, *DA^t^* of testing ends with higher *DA^t^* of training, which is 99.28%. The reason is because testing is based on training results, and so testing performs better.

### 5.3. Parameter Investigation

In this section, we investigate the effects of some important parameters and our observations; we compare the performance of our methods with LFAD [1] with the performance of mitigation accuracy *MA^t^*. Notably, there are eight previous works that concern defense against LFA for both detection and mitigation [1,4,5,8,10,11,12,13], while others [3,6,7,9] only deal with detection. Overall, the study of [1] performs best in terms of defending against LFA with SDN among the eight previous works cited. Therefore, we selected [1] to compare with our method.

#### 5.3.1. The Effects of Time Series

**Figure 15:**
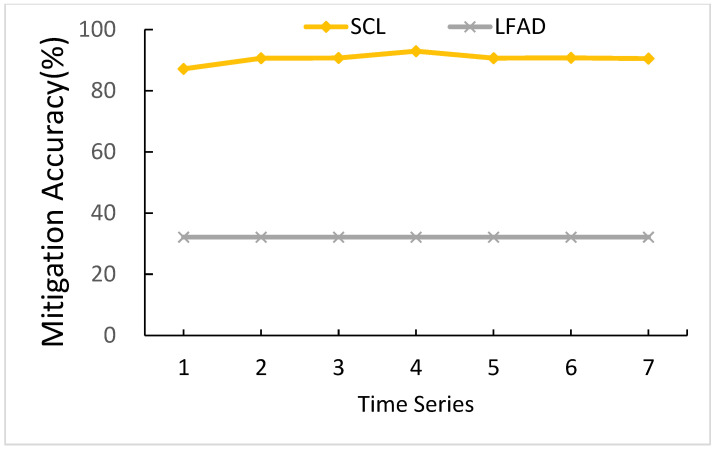
Comparison of different time series.

Time series is the number of consecutive sheets of *U^t^* as input of CNN. Figure 15 shows a comparison of different time series. Except for when the time series is one, when the time series is ranging from two to seven, SCL can maintain a level above 90.64%. As for LFAD, when the time series is ranging from one to seven, LFAD is all the same. Since LFAD does not have the parameter of time series, the *MA^t^* values of LFAD are all the same. In default value (time series = 4), SCL performs better than LFAD by 60.81%.

The reason for SCL’s better performance is because when the time series is one, there is no time series order concept, and *MA^t^* is the lowest. As for when the time series is two to seven, since there is a time series order concept to predict LFA, *MA^t^* values are relatively similar. The highest *MA^t^* is time series equals four, for which the level is 92.95%, so we chose four time series as our parameter.

#### 5.3.2. The effects of the Number of Target Links

**Figure 16:**
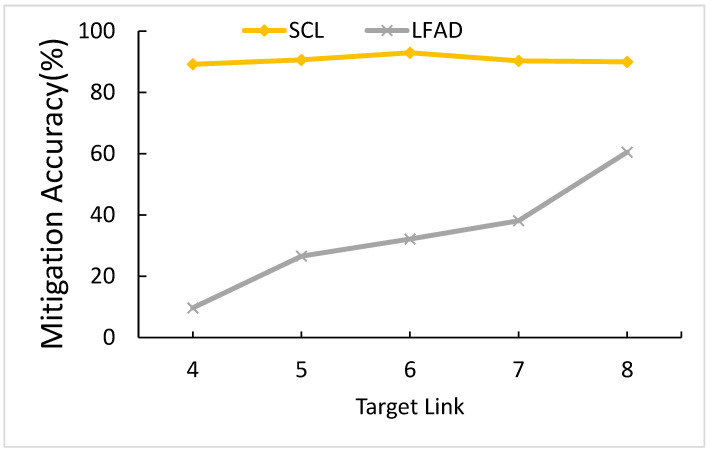
Comparison of the number of input links.

The number of target links is how many numbers of target links the LFA are about to attack. Figure 16 shows a comparison of the number of target links. When the number of target links is ranging from four to eight, SCL can maintain a level above 88.87%, which is more stable than LFAD. As for LFAD, when the number of target links is ranging from four to eight, LFAD increases from 9.68 to 60.53%. Therefore, when the number of target links is ranging from four to eight, the gap between SCL and LFAD is less. In default value (target links = 6), SCL performs better than LFAD by 60.81%.

The reason for SCL performing better is because SCL is predicted by a deep learning method, the deep learning determines if an attack happens according to link utilization *U^t^* to learn the attack flow pattern. The attack flow pattern of the different number of target links directly reflects on *U^t^*, so there is only the small effect for SCL in the different number of target links. On the other hand, the reason for LFAD’s lower performance is that LFAD is to set identify rules on target links, which is to put a sensor on the target links to predict the abnormal situations. The more target links there are, the more sensors there will be and the more accurate LFAD will be. The highest *MA^t^* is six target links, which is 92.95%, so we chose six target links as our parameter.

#### 5.3.3. The Effects of the Number of Input Nodes

**Figure 17:**
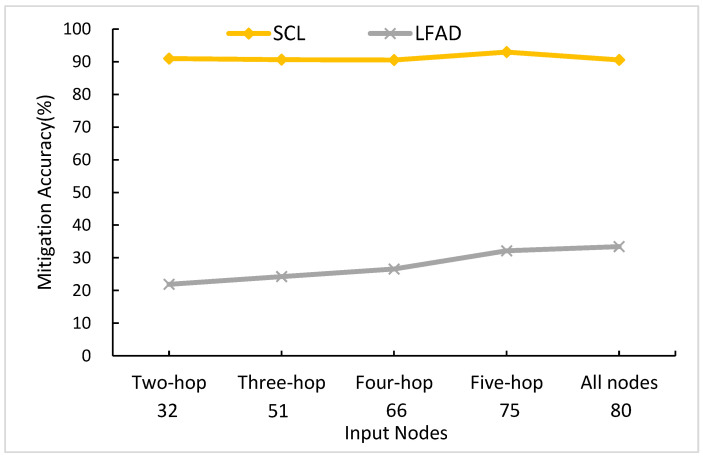
Comparison of the number of target links.

The number of input nodes is the input dimensions. For example, if the number of input nodes is the two-hop, that means the input nodes of *U^t^* are 32 × 32. The hop means, with the target link as the center, the number of hops outward. Two-hop is to make target links as a center and go out according to a distance of two-hop links. As shown in Figure 17, there are five types of input nodes, and the number on each *x*-axis denotes the number of nodes in *U^t^*. Therefore, the input dimensions of each of them are 32 × 32, 51 × 51, 66 × 66, 75 × 75, and 80 × 80, respectively. When the number of input nodes is ranging from two-hop to all nodes, SCL can maintain a level above 90.51%, which is more stable than LFAD. As for LFAD, when the number of input nodes is ranging from two-hop to all nodes, LFAD increases from 21.88 to 33.42%. Therefore, when the number of input nodes is ranging from two-hop to all nodes, the gap between SCL and LFAD is less. 

The reason for this is that SCL learns from *U^t^*; the higher the input nodes are, the more data SCL can get, and the earlier SCL can detect when there is an attack flow from the outer hop. However, the category of fewer input nodes, like two-hop, spends more time on training, and can still achieve the same performance with the types of higher input nodes, like all nodes. As for LFAD, the reason is the more the input nodes are, the more link data LFAD gets to predict if there is an attack. The highest *MA^t^* is five-hop, which maintains a level of 92.95%, so we chose five-hop as our parameter.

#### 5.3.4. The Effects of the Number of Bots

**Figure 18:**
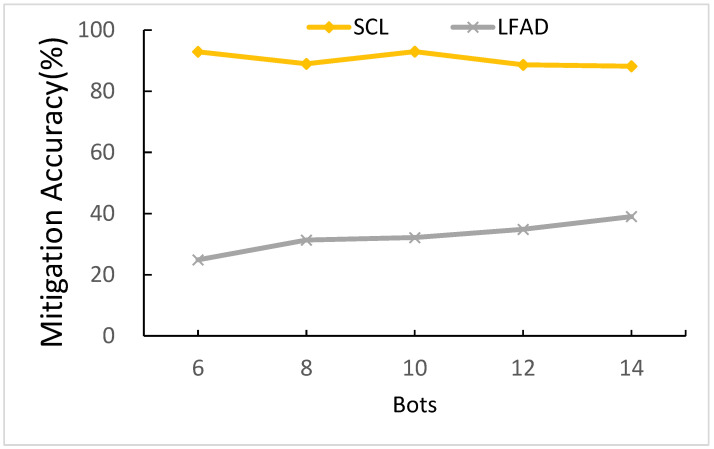
Comparison of the number of bots.

The number of bots is how many bots are used to launch LFA at the same time. Figure 18 shows a comparison of the number of bots. When the number of bots is ranging from 6 to 14, SCL can maintain a level above 88.17%, which is more stable than LFAD. As for LFAD, when the number of bots is ranging from 6 to 14, LFAD increases from 24.87 to 38.99%. Therefore, when the number of bots is ranging from 4 to 16, the gap between SCL and LFAD is less.

The reason for SCL’s better performance is quite the same as Section 5.3.2. Because SCL is predicted by the deep learning method, the deep learning determines if attacks happen according to link utilization *U^t^* to learn the attack flow pattern. The attack flow pattern of the different number of bots directly reflects on *U^t^*, so there is only the small effect for SCL in the different number of bots. As for LFAD, since the more the number of bots is, the more flow traffic there will be, LFAD can thus detect attack easily. The highest *MA^t^* is ten bots, which is equivalent to a level of 92.95%, so we chose ten bots as our parameter.

## 6. Conclusions and Future Work

This study identifies that LFA are difficult to defend against due to (1) the indistinguishability of the changing LFA characteristics and (2) the spatiotemporal attack pattern, leading to the rising attention on developing varying LFA defense methodologies. In light of the previous works that do not provide sufficient performance when addressing these two issues, we propose stacking-based CNN and LSTM deep learning modules to defend against LFA. 

The novelties of SCL are: (a) combining continuous network status as an input to represent “continuous/combination attacking action” and to help CNN operation to extract features of time series attack pattern, (b) applying LSTM to periodically review the current evolved LFA patterns and drop the obsolete ones to ensure the decision accuracy and confidence, (c) stacking System Detector and LFA Mitigator module instead of only one module to couple with LFA detection and mediation at the same time.

The simulation results show that the accuracy of SCL to determine a system under attack or not is 94.38%% and the accuracy of successfully blocking LFA is 92.95%. The accuracy of successfully blocking LFA of SCL is 60.81% higher than that for LFAD. SCL can maintain a level above 88.17% in the different time series, the different numbers of target links, input nodes, and bots, which is more stable than LFAD.

In the future, we will extend our work in the real world to ensure our work still has the expected effectiveness. On the other hand, we will evaluate the performance of new AI architecture, such as seq2seq models [38], and integrate these ideas into our work.

## Figures and Tables

**Table 2:** Used notations.

Notations	Descriptions	Property
*C*	Network capacity matrix *C*$\;=\left[\begin{array}{ccc} c_{1,1} & \ldots & c_{1,z}\\ \vdots & \ddots & \vdots \\ c_{z,1} & \ldots & c_{z,z} \end{array}\right]$, where the *c_i_*_,*j*_ is the link capacity from node *i* to *j*	Input
*TA*	Target area	Input
*M*	Number of target links in the topology	Input
*max^t^*	Maximum threshold of link utilization at time *t*	Input
*min^t^*	Minimum threshold of link utilization at time *t*	Input
*K*	Number of continuous inputs for deep learning	Input
*ε*	When detection accuracy less than *ε* for several times, the training ends. Specifically, $\left(DA^{t}-DA^{t-1}\leq \varepsilon \right)$ and $\left(DA^{t-1}-DA^{t-2}\leq \varepsilon \right)$ and … $\left(DA^{t-D+1}-DA^{t-D}\leq \varepsilon \right)$, the training of System Detector ends	Input
*D*	The accumulated times reaches *D* when $\left(DA^{t}-DA^{t-1}\leq \varepsilon \right)$, the training of System Detector ends	Input
*S^t^*	Network traffic matrix *S^t^*$\;=\left[\begin{array}{ccc} s_{1,1}^{t\;} & \ldots & s_{1,z}^{t\;}\\ \vdots & \ddots & \vdots \\ s_{z,1}^{t\;} & \ldots & s_{z,z}^{t\;} \end{array}\right]$, where the *s_i_*_,*j*_ is the amount of traffic from node *i* to *j* at time *t*	Variable
*U^t^*	Network utilization matrix *U^t^*$\;=\left[\begin{array}{ccc} U_{1,1}^{t\;} & \ldots & U_{1,z}^{t\;}\\ \vdots & \ddots & \vdots \\ U_{z,1}^{t\;} & \ldots & U_{z,z}^{t\;} \end{array}\right]$, where the *U_i_*_,*j*_ is the link utilization from node *i* to *j*. Specifically, $U_{i,j}=\frac{s_{i,j}}{c_{i,j}}$	Variable
*B^t^*	The vector of target links under attack or not at time *t*. Specifically, $B^{t}=\left[b_{1}^{t},b_{2}^{t},b_{3}^{t},\ldots ,b_{M}^{t}\right]$, where $b_{i}^{t}$ is under attack or not in the *i*-th target link at time *t*	Variable
*N^t^*	Number of attacked target links at time *t*. The total number of attacked target links in *B^t^*	Variable
*PAT^t^*	The probability of attacked target links at time *t*. Specifically, $PAT^{t}=\frac{N^{t}}{M}$	Variable
*DTP^t^*	The number of true positive in detection, which means the number of identified attacks and actual attacks	Variable
*DTN^t^*	The number of true negative in detection, which means the number of identified non-attacks and actual non-attacks	Variable
*DFP^t^*	The number of false positive in detection, which means the number of identified attacks but actual non-attacks	Variable
*DFN^t^*	The number of false negative in detection, which means the number of identified non-attacks but actual attacks	Variable
*DA^t^*	Detection accuracy of system under attack or not, which is the performance of System Detector. Specifically, $DA^{t}=\frac{DTP^{t}+DTN^{t}}{DTP^{t}+DFP^{t}+DFN^{t}+DTN^{t}}$	Variable
*MTP^t^*	The number of true positive in mitigation, which means the number of flows should be blocked and actually be blocked	Variable
*MTN^t^*	The number of true negative in mitigation, which means the number of flows should not be blocked and actually not be blocked	Variable
*MFP^t^*	The number of false positive in mitigation, which means the number of flows should be blocked but actually not be blocked	Variable
*MFN^t^*	The number of false negative in mitigation, which means the number of flows should not be blocked but actually be blocked	Variable
*MA^t^*	Mitigation accuracy of successfully blocking LFA, which is the performance of LFA Mitigator module. Specifically, $MA^{t}=\frac{MTP^{t}+MTN^{t}}{MTP^{t}+MFP^{t}+MFN^{t}+MTN^{t}}$	Variable
*NT*	Number of training times	Variable
*ET*	The time that deep learning spends in each training time	Variable
*OTT*	The overall training time in deep learning. Specifically, OTT=NT×ET	Variable
*A2O*	The measure of the relationship between *DA^t^* and *OTT*. Specifically, $A2O={\left(DA^{t}\right)}^{2}/OTT$	Variable
FRR	False rejection rate of system under attack or not, which means it should be recognized as non-attack, but it is determine as an attack. Specifically, $FRR=\frac{DFN^{t}}{DTP^{t}+DFN^{t}}$	Variable
FAR	False acceptance rate of system under attack or not, which means it should be recognized as an attack, but it is determine as non-attack. Specifically, $FAR=\frac{DFP^{t}}{DFP^{t}+DTN^{t}}$	Variable
*P^t^*	The vector of dropping probability of target links at time *t*. Specifically, $P^{t}=\left[p_{1}^{t},p_{2}^{t},p_{3}^{t},\ldots ,p_{M}^{t}\right]$*,* where $p_{i}^{t}$ is the dropping probability in the *i*-th target link at time *t*	Output

**Table 3:** Default values for simulation.

Parameter	Default Value
Number of nodes	80
The way to decide target links	All links get into TA
Link capacity	60 Mbps
Routing method	Shortest path
Normal	**Default Value**
Number of traffic	1–50
Source and destination	Node -> Node
Flow traffic	1–60 Mbps
Lasted time	All the time
Launch interval	Random
Attack	**Default Value**
Number of bots	10
Source and destination	Bot -> Decoy server
Flow traffic	4 Kbps per flow
Launch interval	3 min

**Table 4:** SCL parameter setting.

Parameter	Default Value
Network capacity matrix (C)	Five-hop (75 × 75)
Target area (TA)	Area in the Figure 8
Number of target links (M)	6
Maximum thresholds of link utilization at time t (maxt)	0.6
Minimum thresholds of link utilization at time t (mint)	0.3
Number of continuous inputs for deep learning (K)	4
When detection accuracy less than *ε* for several times, the training ends (*ε*)	002
The accumulated times which $\left(DA^{t}-DA^{t-1}\leq \varepsilon \right)$, the training of System Detector ends (D)	15

**Table 5:** CNN parameter setting.

Parameter	Default Value
1st Convolution Layer	32 filters (shape 8 × 8 × 4), with stride = 4 and ReLU (Rectified Linear Unit) function.
2nd Convolution Layer	64 filters (shape 4 × 4 × 32), with stride = 2 and ReLU function
3rd Convolution Layer	64 filters (shape 3 × 3 × 64), with stride = 2 and ReLU function
Pooling Layer	Max pool function, with stride = 2
Fully Connected layer	Flatten and transform to one-dimension vector (1 × 512)
Convolutions order	Convolution -> Pooling -> Convolution -> Convolution -> Fully Connected

## Data Availability

Not applicable.
